# Modified Poly(Lactic Acid) Epoxy Resin Using Chitosan for Reactive Blending with Epoxidized Natural Rubber: Analysis of Annealing Time

**DOI:** 10.3390/polym14061085

**Published:** 2022-03-08

**Authors:** Thidarat Kanthiya, Krittameth Kiattipornpithak, Nanthicha Thajai, Yuthana Phimolsiripol, Pornchai Rachtanapun, Sarinthip Thanakkasaranee, Noppol Leksawasdi, Nuttapol Tanadchangsaeng, Choncharoen Sawangrat, Pitiwat Wattanachai, Kittisak Jantanasakulwong

**Affiliations:** 1 School of Agro-Industry, Faculty of Agro-Industry, Chiang Mai University, Mae-Hea, Mueang, Chiang Mai 50100, Thailand; thidaratkanthiya05@gmail.com (T.K.); first200294@gmail.com (K.K.); yuthana.p@cmu.ac.th (Y.P.); pornchai.r@cmu.ac.th (P.R.); sarinthip.t@cmu.ac.th (S.T.); noppol.l@cmu.ac.th (N.L.); 2 Faculty of Science, Chiang Mai University, Chiang Mai 50200, Thailand; nanthicha581@gmail.com; 3 Cluster of Agro Bio-Circular-Green Industry, Faculty of Agro-Industry, Chiang Mai University, Chiang Mai 50100, Thailand; 4 College of Biomedical Engineering, Rangsit University, Pathumthani 12000, Thailand; nuttapol.t@rsu.ac.th; 5 Department of Industrial Engineering, Faculty of Engineering, Chiang Mai University, Chiang Mai 50200, Thailand; choncharoen@step.cmu.ac.th; 6 Department of Civil Engineering, Faculty of Engineering, Chiang Mai University, Chiang Mai 50200, Thailand

**Keywords:** crosslinking, compatibility, mechanical property, morphology, crosslink

## Abstract

Poly(lactic acid) was melt-blended with epoxy resin without hardener and chitosan (CTS) to prepare modified PLA (PLAEC). Epoxy resin 5% and CTS 1–20% (wt/wt) were incorporated into PLA during melt mixing. PLAEC was melt-blended with an epoxidized natural rubber (ENR) 80/20 wt. The PLAEC CTS 1% blended with ENR (PLAEC1/ENR) showed a high tensile strength (30 MPa) and elongation at break (7%). The annealing process at 80 °C for 0–15 min maintained a tensile strength of approximately 30 MPa. SEM images of the PLAE/ENR blend showed phase inversion from co-continuous to ENR particle dispersion in the PLA matrix with the addition of CTS, whereas the annealing time reduced the hole sizes of the extracted ENR phase due to the shrinkage of PLA by crystallization. Thermal properties were observed by DSC and a Vicat softening test. The annealing process increased the crystallinity and Vicat softening temperature of the PLAEC1/ENR blend. Reactions of −COOH/epoxy groups and epoxy/−NH_2_ groups occurred during PLAE and PLAEC preparation, respectively. FTIR confirmed the reaction between the −NH_2_ groups of CTS in PLAEC and the epoxy groups of ENR. This reaction increased the mechanical properties, while the annealing process improved the morphology and thermal properties of the blend.

## 1. Introduction

Petroleum-based plastics are a major environmental concern owing to their inability to degrade naturally. This lack of degradation affects organisms living on land and in the sea. Biodegradable polymers, such as polybutylene succinate (PBS) [1], polylactic acid (PLA) [2], polysaccharides [3], carboxymethyl cellulose [4,5,6], carboxymethyl bacterial cellulose [7], CTS [8], carboxymethyl chitosan [9], starch [10,11,12], thermoplastic starch (TPS) [13,14,15], keratin [16], and pectin [17,18], have been widely studied. PLA is a biodegradable aliphatic polyester. It is transparent and has a high modulus and strength comparable to many petroleum-based plastics [19,20], such as polyethylene terephthalate and polystyrene. It can be naturally degraded at a shorter degradation time compared to petroleum-based plastics, such as polyethylene terephthalate, that has a degradation time of 450 years [21]. However, the use of PLA has many limitations owing to the high cost of production as well as unsuitable mechanical properties, such as brittleness, low impact resistance, low elongation at break, and low thermal resistance. However, the use of polymer blends that react with their terminal carboxyl and hydroxyl groups [22,23] can improve the mechanical properties of bioplastics.

Epoxy resin is an intermolecular binding agent containing oxirene rings with a triangular ether structure. The oxygen atom forms a bond with two carbon atoms, thereby vigorously making the oxygen atom of this epoxide structure capable of forming covalent bonds with amine alcohol and carboxylic groups through the ring-opening reaction [23,24]. Studies have reported that epoxy improves the toughness of PLA [25]. The addition of epoxy increased the impact resistance of the PLA and the addition of more epoxy increased the thermal stability [22,23].

Epoxidized natural rubber (ENR) is a soft, highly flexible material with good resilience and high-impact dispersion properties. It can be synthesized by epoxidation of natural rubber molecules to form epoxide groups or oxirane rings on the double bond position of natural rubber; ENR shows higher polarity than natural rubber [26]. The chemical and electron beams are used to form crosslinks in rubbers [27]. ENR presents epoxy bands of FITR at 836 and 870 cm^−1^ [28]. It is resistant to various chemicals and oils, making it resistant to chemical reactions. The properties of epoxidized natural rubber depend on the amount of oxirane groups in the chain of natural rubber [29].

CTS is a natural polymer with outstanding properties, including high mechanical properties, biodegradability, antimicrobial activity, and nontoxicity [30,31,32]. It easily reacts with other chemicals because of the presence of amine groups. Because of its physical and chemical properties, CTS has been used in a wide variety of applications [33,34]. The use of CTS as a compatibilizer between TPS and ENR has been reported; the presence of CTS improves tensile strength and increases the chain length of the polymer [35]. However, reactive blending of PLA, epoxy, CTS, and ENR has not been reported before. PLA shows high mechanical but low thermal resistant properties. Therefore, reactive blending with the induced reactions and crystallinity process is used to develop high thermal resistant PLA. 

In this study, high temperature resistant PLA was developed by melt-blending with epoxy resin, CTS, and ENR with an annealing process at 80 °C for 0–120 min. It has been reported that an annealing temperature of 80 °C induced high crystallinity content in PLA [36]. Epoxy resin was used as a crosslinking agent to improve the toughness of PLA, whereas CTS was selected to enhance the reaction between the amino groups of CTS and epoxy groups of epoxy resin or ENR. The mechanical properties, morphology, thermal properties, crystallinity, and reactions of the PLA/ENR blend were investigated.

## 2. Materials and Methods

### 2.1. Materials

PLA (4043D, density 1.24 g/cc, MFI = 7 g/10 min at 210 °C, MW 100,000 g/mol, NatureWorks LLC, Minnetonka, MN, USA) was acquired from PTT Global Chemical Pub Co., Ltd., Bangkok, Thailand. Epoxy resin (diglycidyl ether of bisphenol) grade A 0302 with the trade name of Easy-resin was purchased from Easy Resin Co., Ltd., Nonthaburi, Thailand. ENR with 25% epoxidation was purchased from Muang Mai Guthrie Public Co., Ltd., Phuket, Thailand. CTS (molecular weight, 500 kDa; deacetylation degree, 85%) and lactic acid (AR grade, 88%) were purchased from Union Science Co., Ltd., Chiang Mai, Thailand. 

### 2.2. Sample Preparation

PLA was melt-blended with epoxy resin, CTS, and ENR using a two-roll mill (PI-140, Pirom-Olarn Co. Ltd., Bangkok, Thailand) at 160 °C for 10 min. It was first melt-blended with 5% epoxy resin (PLAE). CTS was dissolved in distilled water with 2% (weight by volume) of lactic acid in distilled water and then 1–20% CTS was melt-blended with PLAE (PLAEC) to induce compatibility and reaction. PLAE and PLAEC were melt-blended with 20% ENR at 160 °C for 10 min. The code names and compositions of the blends are listed in Table 1. The samples were compressed into sheets using a hot compress at 160 °C for 3 min, followed by quenching in cool water. The samples were annealed at 80 °C for 5, 15, 30, 60, and 120 min to observe the effect of the annealing time.

### 2.3. Tensile Properties

The tensile test was performed following JISK-6251-7 using a tensile tester (Model H1KS, Hounfield Test Equipment, Surrey, UK) at a crosshead speed of 10 mm/min. The bone-shaped samples were prepared as sheets by compression molding at 160 °C for 3 min to study the tensile strength and elongation at break. Five specimens of each sample were observed.

### 2.4. Scanning Electron Microscopy (SEM)

The morphology of the samples was observed using SEM (JSM-IT 300LV model, Tokyo, Japan). The samples were prepared as sheets and broken in liquid nitrogen. The fractured surfaces of the samples were prepared by immersing the samples in toluene at 25 °C for 24 h to study the dispersion structure of the blends. The prepared fracture surface was coated with a thin layer of gold by sputtering (108 Auto/SE sputter coater, Cressington Co., Ltd., Watford, England). The particle sizes of the rubber were calculated using ImageJ software.

### 2.5. Differential Scanning Calorimetry (DSC) 

The samples (8–10 mg) were placed in an alumina pan and analyzed using DSC (823E, Mettler Toledo LLC, Columbus, OH, USA). The second cycle profile was observed with a heating rate of 10 °C/min from 0 to 200 °C. The measurements were conducted under a nitrogen atmosphere to analyze the glass transition temperature (*T_g_*), melting temperature (*T_m_*), and crystallization content of PLA. The crystallinity (%*X_c_*) of PLA was calculated using Equation (1) [37]:

(1) $$\%X_{c}\;=\;\left(\frac{\Delta H_{m}\;-\;\Delta H_{c}}{\omega H_{m}^{0}}\;\right)\;\times \;100$$

where Δ*H_m_* and Δ*H_c_* are the enthalpies of melting and cold crystallization, respectively, and *ω* and Δ$H_{m}^{0}\;$ are the weight fraction of PLA and the melting enthalpy of 100% PLA (93.7 J/g) [37], respectively.

### 2.6. Vicat Softening Temperature (VST) 

Samples with dimensions of 10 mm × 10 mm × 4 mm (width × length × thickness) were prepared using hot compression molding. The samples were heated until a flattened needle penetrated 1 mm into the surface using the ASTM D1525 standard. At least five specimens were measured for each sample.

### 2.7. Fourier-Transform Infrared Spectroscopy (FTIR)

Fourier-transform infrared spectroscopy (FTIR-4700; Jasco Corp., Tokyo, Japan) was used to observe the reactions of the PLAEC/ENR blend. The samples were prepared as sheets by compression molding at 160 °C for 3 min. The FTIR spectrum was measured at 800–4000 cm^−1^ with a resolution of 4 cm^−1^. We expected to detect new vibration bands of C−O and C=O by the occurred reaction.

### 2.8. Statistical Analysis

One-way analysis of variance (ANOVA) was used to analyze results with the Statistical Package for the Social Sciences, SPSS Version 17 (SPSS, Armonk, NY, USA). The differences (*p* < 0.05) were estimated using Duncan’s test.

## 3. Results and Discussion

### 3.1. Reaction Mechanism

**Figure 1:**
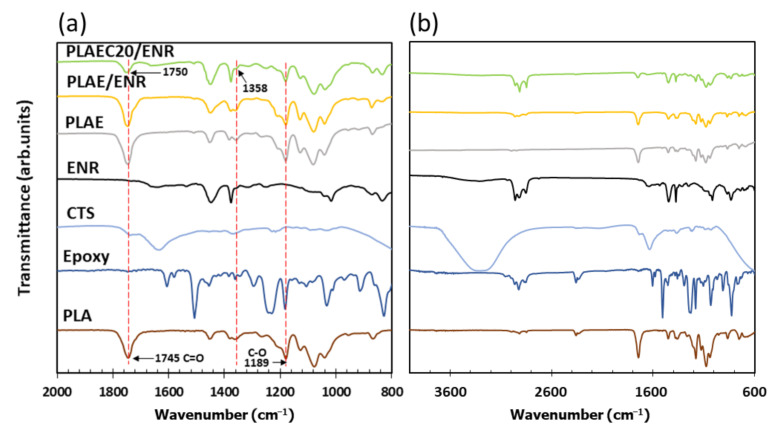
FTIR spectra of PLA, epoxy, CTS, ENR, PLAE, PLAE/ENR, and PLAEC20/ENR at (**a**) 800–2000 cm^−1^ and (**b**) 600–4000 cm^–1^.

**Figure 2:**
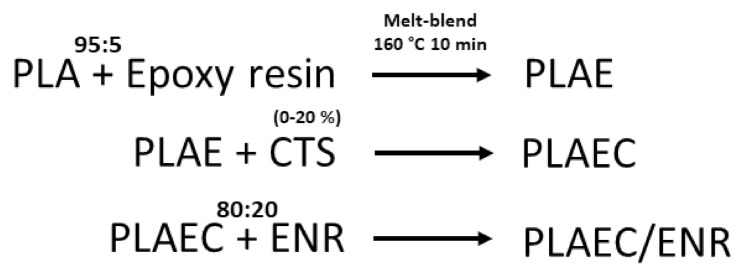
Schematic of sample preparation.

**Figure 3:**
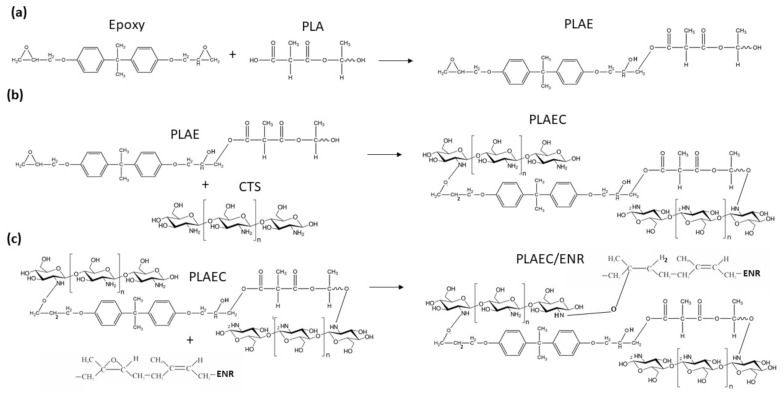
Suggested reactions of (**a**) epoxy and PLA, (**b**) PLAE and CTS, and (**c**) PLAEC and ENR.

Figure 1 shows the FTIR spectra of PLA, epoxy, CTS, ENR, PLAE, PLAE/ENR, and PLAE20/ENR. PLA showed C–O, C–O–C, C=O, and −CH_3_ asymmetry, and −CH_3_ symmetry, at 1080, 1189, 1745, 2995, and 2946 cm^−1^, respectively [38,39]. Epoxy showed characteristic peaks at 914 and 1610 cm^−1^ due to epoxy groups’ absorption and C=C stretching bands of aromatic rings, respectively. The peaks at 1459, 1508, 1581, and 1606 corresponded to C–C stretching vibration of the aromatic ring [40,41]. CTS spectra presented peaks at 1325, 1550, and 1645 cm^−1^ due to the C–N stretching of amide III, N–H bending of amide II, and C=O stretching of amide I, respectively. The bands at 2877 and 2921 cm^−1^ were attributed to C–H asymmetric and symmetric, respectively, whereas 3291–3610 cm^−1^ corresponded to N–H and O–H stretching [42]. ENR exhibited symmetric and asymmetric epoxide bands at 836 and 870 cm^−1^, respectively [28]. The bands at 1377 and 1448 cm^−1^ were attributed to stretching of the −CH_3_ and C–H groups, respectively, whereas bands at 2854, 2917, and 2961 cm^−1^ were due to C–H stretching and −CH_2_ groups [28,43]. The PLA, PLAE, PLAE/ENR, and PLAEC20/ENR samples were normalized using a peak at 1080 cm^−1^ of PLA. PLAEC20/ENR was selected to compare FTIR spectra due to the high intensity of CTS spectra. A schematic of the sample preparation is shown in Figure 2. The PLA blended with epoxy sample showed the main spectra of PLA with the C–C stretching peak of epoxy at 1508 cm^−1^ and epoxy groups at 914 cm^−1^ due to the remaining epoxy resin in PLA through a reaction between PLA and epoxy (Figure 3a). The reaction mechanism between −COOH of PLA and the epoxy groups of epoxy resin was presented in previous research [25]. The PLAE/ENR blend represented combination spectra between PLA and ENR, whereas peaks of epoxy resin at 914 cm^−1^ (epoxy groups) and 1508 cm^−1^ (C–C stretching) were also observed. The PLAEC20/ENR blend showed a shifting C=O peak of PLA from 1745 to 1750 cm^−1^, whereas the epoxy groups of the epoxy resin (914 cm^−1^) peak were not observed. The disappearance of the peak at 914 cm^−1^ indicated the formation of a reaction between NH_2_ groups of CTS and epoxy groups of epoxy resin (Figure 3b). However, in the PLAEC20/ENR blend, epoxy peaks of ENR appeared at 836 and 870 cm^−1^ due to the unreacted epoxy groups inside the ENR rubber particles. The reaction between NH_2_ groups of CTS and epoxy groups of ENR was also apparent (Figure 3c). The reaction between NH_2_ groups of CTS and epoxy groups of ENR was confirmed in previous research [28]. The reaction between −COOH groups and NH_2_ groups of CTS has also been previously reported [44]. It was confirmed that the epoxy resin reacted with −COOH of PLA and NH_2_ groups of CTS, whereas the interfacial reaction of the PLAEC20/ENR blend was due to a reaction between the NH_2_ groups of CTS and epoxy groups of ENR (Figure 3c). 

### 3.2. Mechanical Properties

**Figure 4:**
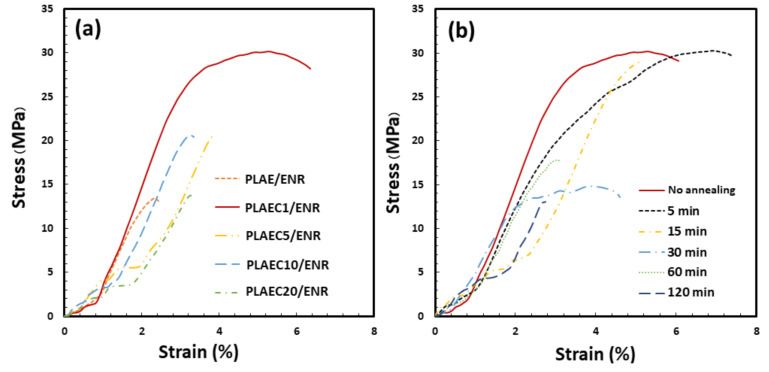
Tensile strength and elongation at break of (**a**) PLA and PLAEC/ENR blend with different CTS contents and (**b**) PLAEC1/ENR blend with different annealing times (0–120 min) at 80 °C.

PLA was melt-blended with 5% epoxy followed by 1–20% CTS to prepare PLAEC, which was then melt-blended with ENR (80/20 wt/wt). Tensile properties were observed using a tensile tester as shown in Figure 4a,b. Poly(lactic acid) blended with 5% epoxy (PLAE) and then blended with ENR was used as a control. The tensile strength (TS) and elongation at break (EB) of the PLAE/ENR were 13 MPa and 2%, respectively, and increased significantly with the addition of 1% CTS to 30 MPa and 6%, respectively (Table 2). The PLAEC/ENR with high CTS content showed a decrease in TS and EB compared with the PLAEC1/ENR blend. The improvement in the tensile properties of PLAEC/ENR was due to the enhanced interfacial crosslink [11] between PLAEC and ENR through CTS. CTS reacts with ENR, as previously reported [45]. The tensile property reduction of the PLAEC/ENR blend with CTS 5–20% indicated an excessive amount of CTS, which increased crosslinking inside the ENR phase and incompatibility between PLA and ENR. Owing to the high tensile property improvement in PLAEC1/ENR, it was annealed at 80 °C for 5–120 min to analyze the effect of annealing times on this property. Annealing at 5 min showed TS and EB at 30 MPa and 8%, respectively, whereas annealing at 15 min resulted in a TS and EB of 28 MPa and 5%, respectively (Figure 4b). The annealing time of 30–120 min decreased the TS and EB of the blends because of the high crystal formation at the thin-layer ligament of PLA between the ENR particles, which easily broke during the tensile test. The high tensile strength of 60 min annealing sample vs. the 30 min annealing sample was due to the suitable network structure of the thin-layer ligament of PLA, whereas the 120 min annealing sample was easily cracked by stress cracking due to the degradation of PLA with long time annealing.

### 3.3. Morphology

**Figure 5:**
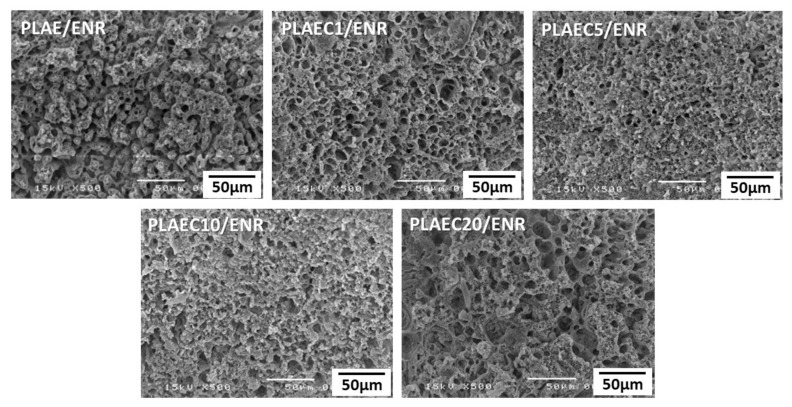
SEM micrographs of PLA and PLAEC/ENR blend with different CTS contents.

**Figure 6:**
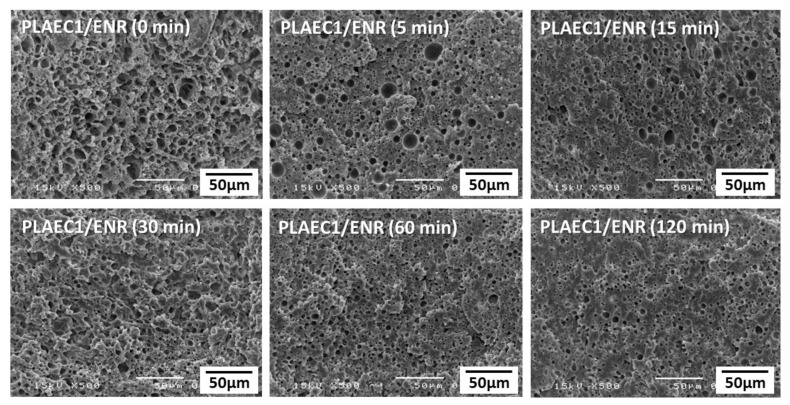
SEM micrographs of the PLAEC1/ENR blend after annealing (0–120 min) at 80 °C.

The SEM micrograph provided additional information on the compatibility of the polymer composite. The ENR phase was extracted from the PLA matrix phase by immersion in toluene. Figure 5 shows the SEM images of the PLAEC/ENR blends with 1%, 5%, 10%, and 20% CTS. The holes on the fracture surface of the sample presented the distribution of the ENR phase. The fractured surface of the PLAE/ENR showed the rubber removed out of the ENR continuous phase with some holes of the ENR particles in the PLAE phase. Phase inversion from co-continuous to the ENR particle dispersion in the PLAEC matrix was observed in PLAEC1/ENR. PLAEC5/ENR and PLAEC10/ENR showed ENR with small particle sizes in the PLAEC matrix, whereas a large ENR continuous phase was observed in the PLAEC20/ENR because of the excessive amount of CTS crosslinking in the ENR phase [45]. Smaller pore sizes of PLAEC5/ENR and PLAEC10/ENR vs. PLAEC1/ENR and PLAEC20/ENR was due to higher interfacial adhesion via CTS reaction with epoxy groups of PLAE and epoxy resin. The morphology of the PLAEC1/ENR blend with and without annealing at 80 °C is shown in Figure 6. The morphology of the PLAEC1/ENR blend annealed for 5 min showed a flat fracture surface with holes of the extracted ENR rubber particles of sizes of approximately 10 µm. The diameter of the rubber particles decreased (3.7 µm) with annealing time due to the shrinkage of the PLA phase by recrystallization of the PLA structure. ENR rubber is a soft material that is compressed by shrinkage of the PLA phase during the annealing process. Small ligaments of the PLA phase between the ENR rubber particles were formed and easily broke because of the high crystal formation during long-term annealing. This indicated that CTS in the PLAEC reacted with ENR, which improved the interfacial adhesion and morphology of the PLAEC/ENR blend. The optimum CTS content was 1%, which improved the fine morphology of the PLAEC/ENR blend and increased the tensile properties. The annealing time induced shrinkage of the PLA phase by recrystallization.

### 3.4. Thermal Properties

**Figure 7:**
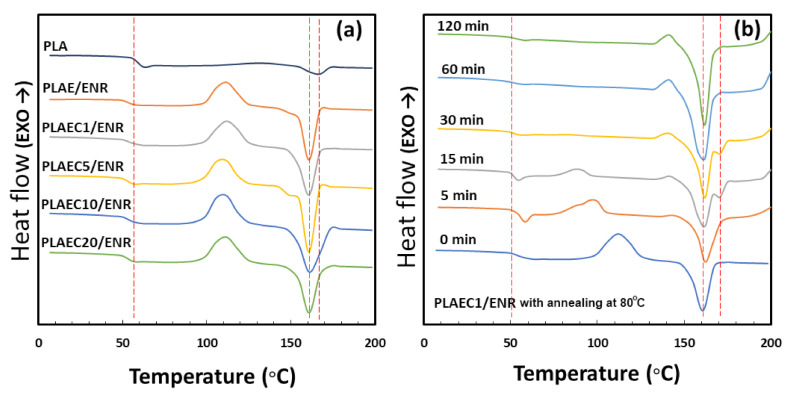
DSC curves of (**a**) second scan of PLA, PLAE/ENR, and PLAEC/ENR with 1–20% CTS and (**b**) first scan of PLAEC1/ENR with annealing (0–120 min) at 80 °C.

Figure 7a shows the second scan DSC curves of PLA and the PLAEC/ENR blend with different CTS contents. PLA and PLAE/ENR showed *T_g_* values of 60 °C and 51 °C, and *T_m_* was 167 °C and 162 °C, respectively (Table 2). The *T_m_* of PLA (167 °C) decreased to 161 °C in the PLAE/ENR and PLAEC/ENR blends. In the PLAE/ENR and PLAEC/ENR blends, recrystallization occurred during the second scan of the DSC measurement at 115 °C, but not in neat PLA. The PLAE/ENR and PLAEC/ENR blends showed a decrease in *T_g_* compared with PLA because of the partial miscibility of the PLA/epoxy blend and the plasticizer effect of epoxy [25]. The decrease in *T_m_* and recrystallization of PLAE/ENR and PLAEC/ENR compared with PLA suggested the small crystal size of PLA due to the nucleating effect of the ENR phase as a nucleating agent for PLA crystallization. The reduction of *T_m_* due to the small crystal size of PLA has been previously reported [46]. Figure 7b shows the first scan DSC curves of PLAEC1/ENR at an annealing temperature of 80 °C for 0–120 min. Annealing samples at 80 °C for 0–120 min showed *T_g_* and *T_m_* values of 51 °C and 162 °C, respectively. Two *T_m_* values were observed at annealing times of 15 and 30 min due to the formation of two PLA crystals [47]. Recrystallization occurred at an annealing time of 0–15 min due to the formation of crystals during the first scan, which is related to incomplete crystallization [48]. Recrystallization was not observed in the annealing time of 30–120 min due to the high crystal content without a nucleation crystal during the first heating scan. The annealing time at 80 °C increased the crystal content of the PLAEC1/ENR blend from 4.9% (0 min) to 36.6% (120 min) (Table 3). ENR particles induced the nucleation rate of the PLAEC/ENR blends, whereas the annealing time increased the crystal content of the PLAEC1/ENR blend.

### 3.5. Vicat Softening Temperature (VST)

**Figure 8:**
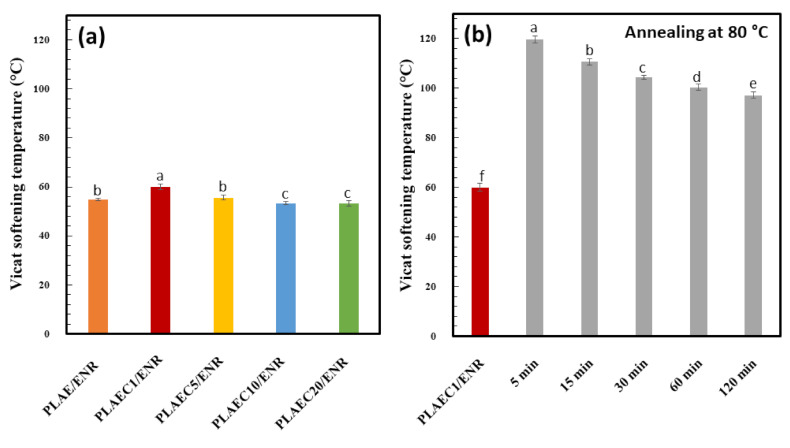
Vicat softening temperature of (**a**) PLAE/ENR blend with 0.5–20% CTS and (**b**) PLAEC1/ENR blend with different annealing times (0–120 min) at 80 °C. Means with different lowercase letters were significantly different (*p* < 0.05).

VST measurements were used to observe the thermal distortion of the polymer. The VST of PLAE/ENR was 55 °C, whereas that of PLAEC/ENR with 1, 5, 10, and 20% CTS were 60, 56, 53, and 53 °C, respectively (Figure 8a). The PLAEC1/ENR showed the highest VST at 60 °C due to its fine morphology, interfacial reaction, and high degree of crystallinity (5%) (Table 3). The increase in CTS resulted in a higher degree of branch structure of molecular chains, which increased the chain distance and decreased the VST of the blends [49]. PLACE/ENR was annealed at 80 °C for 5–120 min. The annealing for 5 min increased the VST to 120 °C; then, the VST decreased with annealing time to 100 °C at 120 min (Figure 8b). The enhanced VST with annealing time of the PLAEC/ENR blends was due to the high percentage of crystallinity (10–37%). An increase in VST by enhancing the crystallinity has been reported [49]. The decrease in VST with an annealing time of 15–120 min was due to the shrinkage of the PLA phase by crystal formation, ENR compression, distortion of PLA by back pressure from the compressed ENR phase, and high softening of ENR at elevated temperature with long-time annealing.

## 4. Conclusions

PLA blends with epoxy resin, CTS, and ENR were successfully developed with improved morphology and mechanical properties. The tensile strength of PLAEC/ENR blend increased with CTS 1%, whereas 5–20% CTS reduced it owing to the excessive amount of CTS and crosslinking inside the ENR phase. The annealing process at 80 °C for longer than 15 min reduced the tensile strength because of the brittleness of the high crystal ligament of PLA between the ENR particles. The morphology of PLAE/ENR blend showed a co-continuous morphology. Phase inversion occurred in ENR rubber particles dispersed in the PLA matrix with CTS addition. The annealing process at 80 °C reduced the pore sizes of the ENR due to shrinkage of the PLA phase by crystallization. DSC showed a reduction in the *T_g_* of PLA with the addition of epoxy, whereas the PLAEC/ENR blend reduced the *T_m_* of PLA. The annealing process at 80 °C increased the crystallinity of the PLAEC1/ENR blend with annealing time. The annealing process at 80 °C for 5–120 min improved the thermal stability of the blend. FTIR confirmed that the −COOH of PLA reacted with epoxy groups of epoxy resin, whereas the −NH groups of CTS reacted with epoxy groups of epoxy resin and ENR. These reactions increased the mechanical properties, while annealing at 80 °C improved the morphology and thermal properties of the blends. The PLAEC/ENR blend with improved properties has the potential for use in packaging, medical, and agricultural applications. 

## Figures and Tables

**Table 1:** Composition of PLAE blending with CTS and ENR.

Sample	Composition (wt/wt%)		
	PLA/Epoxy Resin	CTS	ENR
PLAE/ENR	80	-	20
PLAEC1/ENR	79	1	20
PLAEC5/ENR	75	5	20
PLAEC10/ENR	70	10	20
PLAEC20/ENR	60	20	20

**Table 2:** Tensile properties of PLA, epoxy resin, CTS, and ENR blend, and the PLAEC1/ENR blend with annealing times of 0–120 min.

Sample	Tensile Strength (MPa)	Elongation at Break (%)	Young’s Modulus (MPa)
PLAE/ENR	13.6 ± 0.6 ^a^	2.4 ± 0.1 ^a^	895.64 ± 11.3 ^b^
PLAEC1/ENR	30.0 ± 0.2 ^c^	6.0 ± 0.2 ^d^	1088.26 ± 5.9 ^a^
PLAEC5/ENR	20.8 ± 0.8 ^b^	3.8 ± 0.1 ^c^	620.30 ± 10.3 ^e^
PLAEC10/ENR	20.2 ± 0.3 ^b^	3.3 ± 0.1 ^b^	820.27 ± 2.4 ^c^
PLAEC20/ENR	16.6 ± 0.5 ^a^	3.3 ± 0.1 ^b^	664.82 ± 1.5 ^d^
No annealing	29.9 ± 0.2 ^a^	6.0 ± 0.1 ^b^	1187.63 ± 2.3 ^a^
5 min	30.1 ± 0.1 ^a^	7.3 ± 0.1 ^a^	892.22 ± 1.9 ^b^
15 min	28.9 ± 0.2 ^b^	5.1 ± 0.1 ^c^	892.01 ± 1.5 ^b^
30 min	14.7 ± 0.1 ^d^	4.6 ± 0.2 ^d^	785.48 ± 1.1 ^e^
60 min	17.3 ± 0.5 ^e^	2.9 ± 0.2 ^e^	864.09 ± 6.7 ^c^
120 min	12.9 ± 0.1 ^f^	2.7 ± 0.1 ^f^	379.08 ± 1.5 ^f^

The mean values indicated by the different lowercase superscript letters are significantly different (*p* < 0.05), *n* = 5.

**Table 3:** Second scan DSC results of PLA, PLAE/ENR, and PLAEC/ENR blend with 1–20% CTS, and first scan DSC results of PLAEC1/ENR after annealing (0–120 min) at 80 °C.

Sample	*T_g_* (°C)	*T_m_* (°C)	Δ*H_m_* (J/g)	Δ*H_c_* (J/g)	*X_c_* (%)
PLA	60	167	4.9	6.1	1.3
PLAE/ENR	51	162	35.1	32.3	2.9
PLAEC1/ENR	51	161	31.3	25.9	5.1
PLAEC5/ENR	52	161	30.1	28.9	1.4
PLAEC10/ENR	51	161	31.7	30.3	1.9
PLAEC20/ENR	52	162	29.0	28.8	2.2
PLAEC1/ENR 0 min	51	161	32.8	28.1	4.9
PLAEC1/ENR 5 min	51	164	27.7	16.9	11.5
PLAEC1/ENR 15 min	51	162	37.0	6.3	31.9
PLAEC1/ENR 30 min	51	162	30.5	2.4	30.1
PLAEC1/ENR 60 min	51	161	36.6	3.6	35.3
PLAEC1/ENR 120 min	51	161	38.2	3.9	36.6

## Data Availability

The data presented in this study are available upon request from the corresponding author.

## Abbreviations

CTS, chitosan; DSC, differential scanning calorimetry; ENR, epoxidized natural rubber; FTIR, Fourier-transform infrared spectroscopy; PLA, poly(lactic acid); SEM, scanning electron microscopy; PLAE, poly(lactic acid) blend with epoxy resin; PLAEC, poly(lactic acid) blend with epoxy resin and chitosan; *T_g_*, glass transition temperature; *T_m_*, melting temperature; VST, Vicat softening temperature.
